# The guardian of the genome meets immunotherapy: p53-based strategies

**DOI:** 10.3389/fimmu.2026.1762679

**Published:** 2026-03-10

**Authors:** Tatyana A. Savostyanova, Julia A. Lopatnikova, Sergey V. Sennikov

**Affiliations:** 1 Laboratory of Molecular Immunology, Research Institute of Fundamental and Clinical Immunology, Novosibirsk, Russia; 2 Department of Natural Sciences, Novosibirsk State University, Novosibirsk, Russia; 3 Institute of Medicine and Medical Technologies, Department of Immunology, Novosibirsk State University, Novosibirsk, Russia

**Keywords:** adoptive TCR-T therapy, gene replacement therapy, p53 neoantigens, p53-based vaccines, TP53 mutations, tumor suppressor protein p53

## Abstract

Originally reported as an oncogene and currently known to be a major “genome guardian”, the p53 protein remains one of the most explored transcription factors, exhibiting variety of functions both within transcription regulation and beyond. Given that p53 dysfunction contributes to the majority of human cancers, understanding its regulatory mechanisms and therapeutic potential remains a primary research focus. This review addresses the key aspects of p53 regulation and functionality, analyses its role in tumor evolution, and provides a comprehensive analysis of current and emerging therapeutic strategies targeting the p53, with particular emphasis on immunotherapy approaches.

## 1 Introduction

The tumor suppressor protein p53 serves as a central hub in cellular responses to diverse types of stress and prevents malignant transformation through regulation of cell cycle, apoptosis, senescence, DNA repair, and metabolic reprogramming. Dysregulation of the p53 pathway profoundly affects the majority of human cancers, and more than half of tumors are driven by mutations in the TP53 gene. Beyond its traditional cell housekeeping functions, evidence gathered over the past decade has positioned p53 to be involved in antitumor immunity by modulating both innate and adaptive immune responses, regulating the expression of immune checkpoints, and influencing the immunogenicity of tumor cells.

A particularly compelling aspect of p53 biology in the context of cancer immunotherapy arises from its role as a source of tumor-specific neoantigens caused by TP53 mutations, as well as of tumor-associated antigens generated due to over-accumulation of wild-type p53 in tumor cells, thereby escaping tolerance and enabling T-cell recognition. Unlike private neoantigens, TP53 hotspot mutation landscape (e.g., R175H, Y220C, R273H, G245S) is shared across patients and tumor types, making p53-derived peptides attractive public neoantigens for off-the-shelf immunotherapies. Advances in peptide- and mRNA-based therapeutics and adoptive TCR-engineered T-cell technologies have transformed p53-targeted immunotherapy concepts into clinically relevant strategies.

Although p53-based immunotherapy has proven to be safe and capable of inducing antigen-specific T-cell reactivity in early-phase clinical trials, objective tumor responses remain infrequent and rarely durable. In this review we examine the relationship between p53 status and tumor evolution, comprehensively analyze the spectrum of immunotherapeutic platforms currently exploiting p53 as a target, and discuss key barriers and future directions for translating p53-targeted immunotherapy into effective clinical practice.

## 2 Key aspects of p53 function and regulation

The p53 tumor suppressor plays a critical role in maintaining cellular homeostasis, by orchestrating responses to different types of stress and preventing malignant transformation (1, 2). The full-length p53 protein comprises transcriptional activation domain (TAD, residues 1-63) located on N-terminal end, followed by a proline-rich region (PRR), coordinating zinc central DNA-binding domain (DBD, residues 94-292), oligomerization domain (OD, residues 307–355) and C-terminal regulatory domain (OD, residues 356–393) (3). Notably, TAD and OD domains feature low thermodynamic stability and are naturally disordered under physiological conditions (3–5). This structural plasticity elucidates the diverse biological outcomes of p53 activity that require dynamic communications with a large number of partner proteins, making p53 a hub adjuster of multiple cellular regulatory networks (6–8).

**Figure 1:**
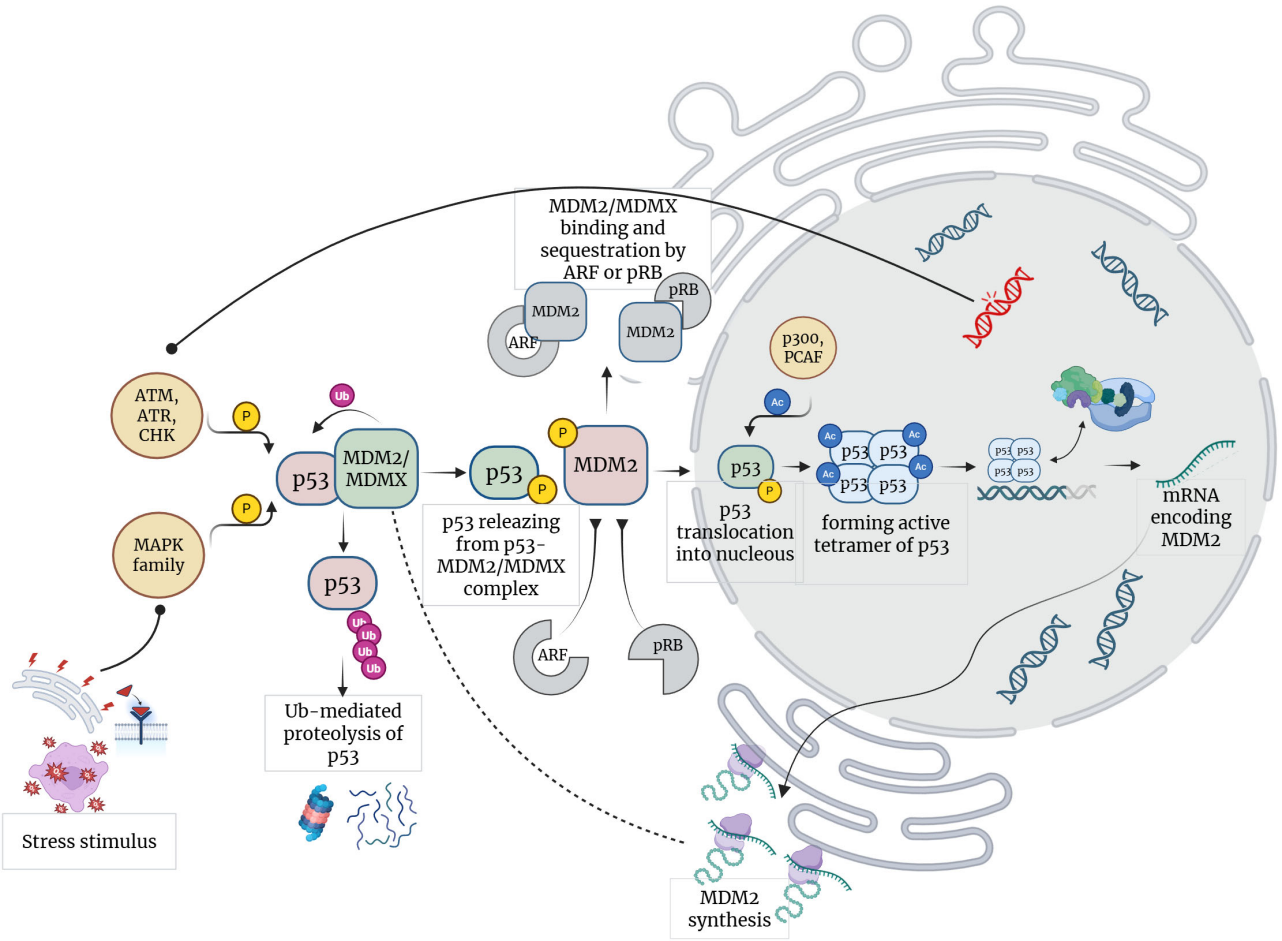
Feedback loops and signaling pathways in p53 regulation. Created in https://BioRender.com.

Normally, cells maintain a modest level of p53 through a balance between its synthesis and degradation, while changes in p53 activity are primarily determined by post-translational modifications (**Figure 1**) (9). Particularly, Mdm2 is determined as a direct negative regulator of p53 via feedback loop, where expression MDM2 is induced by p53 (10, 11). Crucial role of p53–Mdm2 axis is highlighted by embryonic lethality of MDM-/- mice, which is rescued by simultaneous elimination of p53 (12). Noteworthy, a significant part of cellular p53 exists in complex with Mdm2, which serves to directly mediate its inhibitory effects (13, 14). Binding of Mdm2 to TAD residues stabilizes p53 in a conformation that lacks reactivity to partner proteins (15). Mdm2 also functions as a p53-specific E3 ubiquitin ligase (10, 16); however, since p53 mutants deficient in residues 333–353 critical for forming a contact with Mdm2, still undergo proteasomal processing, the precise contribution of Mdm2 in ubiquitin-mediated p53 degradation remains unclear (17–19). Concurrently, in nucleus Mdm2 is essential to decrease activated p53 levels, where ubiquitin tagging facilitates p53 translocation by nuclear export machinery (10, 20). MDMX, another p53 regulator and structural homologue of MDM2, lacks ubiquitin ligase activity and functions as a sequestering factor by sterically impeding p53 interactions (21).

Under stress conditions, p53 activity is primarily modulated by posttranslational modifications within the TAD domain (9, 22, 23). Among them, phosphorylation of Thr18 releases p53 from MDM2/MDMX-p53-complexes and restores the conformation adaptivity of TAD allowing p53 to interact with partner proteins (24). Protein kinases activated by various stress stimuli, including members of the MAPK family (JNK1-3, ERK1-2, p38 MAPK) and DNA damage response kinases (ATR, ATM, CHK1, and CHK2), target the TAD domain, mediating positive upstream regulation of the p53 pathway (25, 26). Given that TAD contains nine phosphorylation sites, diverse kinase cascades generate distinct modification patterns, which underlies the modulation of p53 affinity for competing partner proteins and result in specific p53-dependent transcriptional programs (23, 24, 26). Further, acetylation of the C-terminal end by nuclear acetyltransferases (p300 and PCAF) enables p53 transcriptional activity via exposure of the DNA binding domain, which binds to p53-response elements, presented palindromic motifs 5′-RRRCWWGYYY-3′ or 5′-WGYYYRRRCW-3′ linked by a spacer of 0–13 nucleotides (27, 28).

The pathways driven by p53-mediated transactivation of target genes largely depends on cellular context and stress trigger (1, 6, 29) and can be resolved either by cell cycle arrest, cellular senescence, differentiation or apoptosis (29–32). Interestingly, but p53 has also been shown to enhance the survival of cells exposed to specific stress conditions (33). The range of targets one’s expression p53 affects is broad and has been comprehensively reviewed (32, 34). Among them, p21 prevents G1-S transition by allosterically blocking the formation of CDK2, CDK1, and CDK4/6 complexes with cyclins and inhibits DNA synthesis in S-phase via competing with DNA polymerase processivity factors for PCNA binding sites (35). The p53 involvement in apoptosis is largely depends on its transcriptional upregulation of Bax protein, which disrupts mitochondrial membrane integrity, and pro-apoptotic factors PUMA and NOXA, which promote Bax and Bak activity, releasing them from complexes with anti-apoptotic members of the Bcl-2 family (36).

Additionally, p53 modulates energy metabolism and antioxidant function through upregulation of glycolysis regulatory phosphatase TIGAR, glutaminase GLS2 and glutathione peroxidase GPX1 (37) and suppression of cystine/glutamate antiporter SLC7A11 (38). p53-mediated transcriptional repression is achieved via p53 interaction with transcription factors, including TBP, SP1 and NF-Y (39), or by binding to p53-response elements with motifs slightly different from the consensus (28). The large number of such non-canonical sites are found in LINE1 elements, underscoring the p53 role as a genome guardian (40).

## 3 Role of p53 in cancer evolution

Since its discovery in 1979, the TP53 gene has been recognized as the most commonly mutable gene in human malignancies, with alterations detected in nearly half of all cancers, ranging from 10% to 90% for specific nosologies (41, 42). The mutational landscape of TP53 is remarkably diverse and over 7,000 unique mutations are catalogued in cancer databases (42).

Among them, nine hotspot mutations are found in the DNA-binding domain (R175H, R248Q, R273H, R248W, R273C, R282W, G245S, R249S, Y220C), accounting for up to 30% of all non-synonymous single nucleotide TP53 variants (nSNVs) (43). Interestingly, these sites exhibit no common arrangement pattern within the p53 protein structure, where residues R248 and R273 directly contact DNA, R249 and R282 lie outside the DNA-binding interface, R175 is adjacent to the zinc-coordinating site, whereas G245 and Y220 are distal from all the binding interfaces from all the binding interfaces (44, 45). Moreover, homology-based protein structure prediction analysis using VIPUR algorithm didn’t reveal any trends in the conformation stability of p53 mutants, with most of the substitutions being interpreted as structurally neutral (45, 46). Mechanistically, seven from nine most frequent SNVs affect arginine residues, encoded by CG* codons, and allocate at constitutive methylated CpG sites within TP53. Methylated cytosine is prone to spontaneous deamination yielding characteristic C→T/G→A transitions (46) and since the basal transcription of TP53 is modest, such events are potentially to be missed by the transcription coupled repair system (47).

Mutant p53 variants display the dysfunctional phenotype (44–46) characterized by altered DNA-binding affinity, non-canonical protein-protein interactions, and, rarely, a complete loss of transcriptional activity (48), resulting in compromised regulation of p53-dependent pathways (49). Indeed, various types of cell lines bearing TP53 mutations exhibit undermined expression of p21, as well as PUMA and Bax proteins (50, 51). Since p21 is crucial factor halting cell cycle progression to enable DNA repair, its insufficient activity at checkpoints coupled with resistance to apoptosis promotes consolidation of genetic aberrations, rendering genomic instability (35, 52).

Missense p53 mutants share a significant intracellular accumulation levels, however the underlying mechanism, whether it is inability of mutants to induce MDM2 or their increased resistance to degradation, is incompletely realized (10, 53, 54). Indeed, normal cells subjected to short-term radiation undergo oscillatory behavior of p53 concentration, while prolonged exposure leads to continual increase in p53 level which is not followed by period of degradation. Given the increased p53 levels observed in a wide range of malignancies, independently of TP53 mutation status, the involvement of constant stress affecting tumor cells seems reasonable in driving p53 resistance to degradation (11, 53, 54).

**Figure 2:**
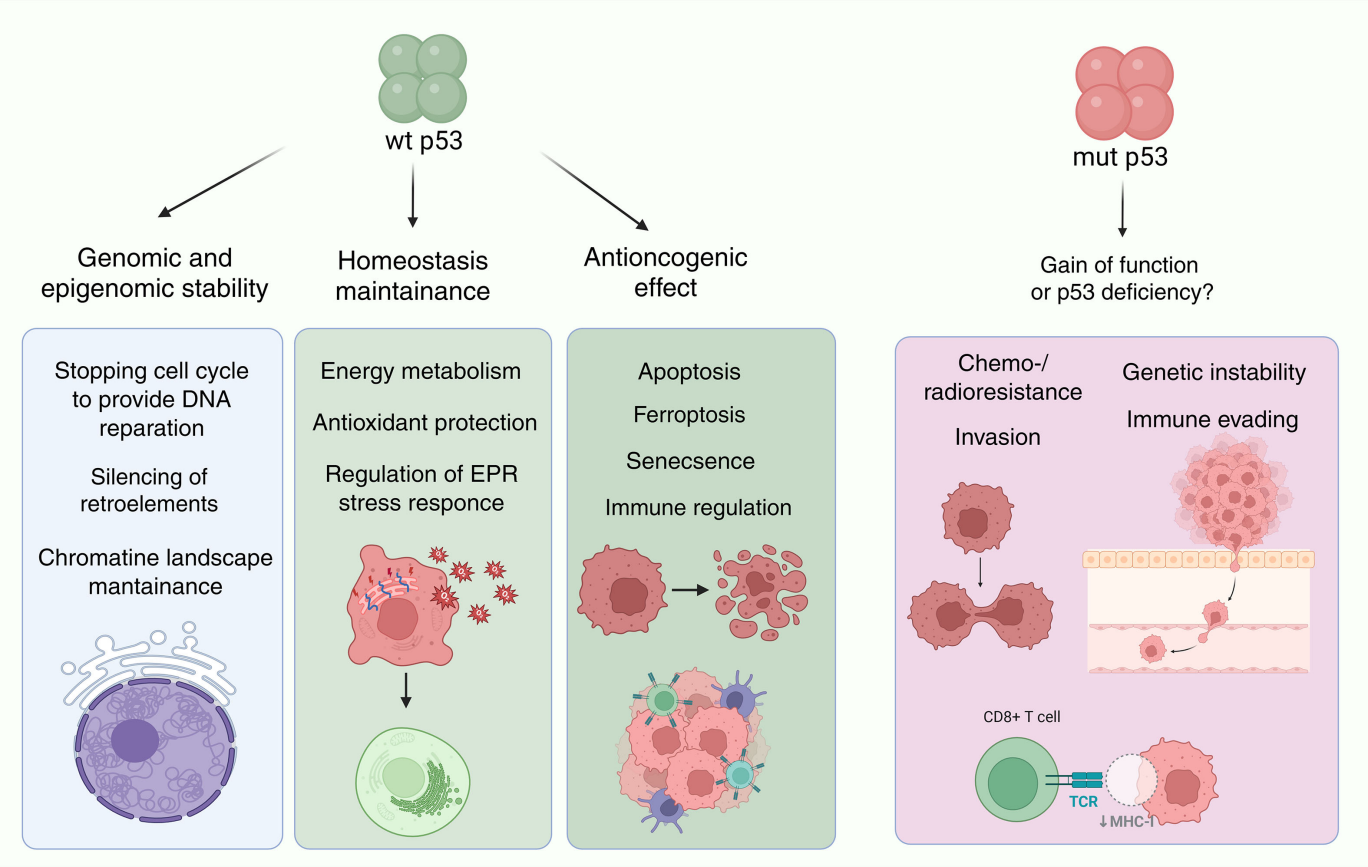
The role of p53 in cancer suppression and tumor evolution. p53 supports chromatin landscape stability by transcriptionally regulating or directly interacting with proteins involved in DNA repair (CDKN1A, BRCA1, XPC, MLH1, PMS2, GADD45A, DDB2), chromatin remodeling (e.g., SMARCA4, ARID1A), epigenetic modification (DNMT1, EZH2, LSD1) and directly suppressing transcription of LINE-1 elements, thus maintaining chromatin integrity and genomic stability. p53 involves in internal and external apoptotic pathways by activating pro-apoptotic genes BAX, PUMA, NOXA and APAF1, which promote cytochrome c releasing from mitochondria and caspase-3 activation, and by upregulating plasma membrane death receptor Fas and DR5. p53 also contributes to ferroptosis by activating GLS2 and repressing SLC7A11, which reduce glutathione synthesis and promote lipid peroxidation. Concurrently, p53 could enhances antioxidant defense via upregulation of GPX1 and SOD2, which neutralize reactive oxygen species. Axes p53/p21 and p16/Rb synergize to block cell cycle and induce senescence, preventing proliferation of damaged cells. p53 abrogates immune checkpoint-mediated immunosuppression via up-regulation of miR-34 and miR-200 family, which directly interact with PD-L1 mRNA. Created in https://BioRender.com.

A key question concerning mutant p53 proteins - whether they function as active oncogenes directly promoting cancer progression - is actively disputed in the literature/lacks consensus among the scientific community (**Figure 2**). Although cell lines bearing mutant TP53 exhibit increased resistance to apoptosis, enhanced potential for epithelial-mesenchymal transition, migration, and chemoresistance - that may be interpreted as gain-of-function (GOF) effects – it remains ambiguous to what extent these phenotypes are driven by ongoing activity of mutant p53 or rather reflect secondary effects of genetic, epigenetic, and metabolic alterations caused by p53 functional deficiency (2, 55–58).

It is now established that genetic changes initially occur in one TP53 allele and render oncogenic effect only if the second wild-type allele is lost, which underscored by monoclonality of cancer cells in terms of p53 mutation profile (59, 60). Tumors harboring mutant TP53 display remarkable nosological heterogeneity (42, 43), with the preference for specific mutation largely dictated by the genetical context (such as methylated CpG-enriched regions), rather than functionality (46, 58). The last is highlighted by the fact that CRISPR-mediated deletion of TP53 in 391 human cancer cell lines, covering 158 distinct mutant p53 variants, had no impact on cell growth or survival (57). In contrast, CRISPR targeting mutant KRAS or BRAF markedly inhibited proliferation of cell lines with corresponding proteins. Similarly, *in vivo* deletion of mutant TP53 did not reduce tumor growth or metastasis of both xenograft and immunocompetent murine cancer models. These findings, along with a high mutational burden found in p53-deficient malignancies, lead to inference that the loss of normal p53 functional activity represents an oncogenic driver in itself, enabling accumulation of genetic, epigenetic and metabolic abnormalities, which in turn constitutes the basis for tumor evolution (57, 61).

## 4 p53 as a therapeutic target

The common incidence of p53 aberrations and its fundamental status as oncogenic driver events mark out p53 one of the most inspiring target in oncology, creating both a therapeutic challenge and opportunity in cancer treatment. This results in a remarkable diversity of clinical modalities ranging from pharmacological modulators of mutant p53 proteins to gene replacement methods aimed at restoring wild-type p53 activity, as well as multiple immunotherapeutic strategies including innovative adaptive cell therapy approaches designed to selectively eliminate p53-dysfunctional cells.

### 4.1 Small drugs

**Figure 3:**
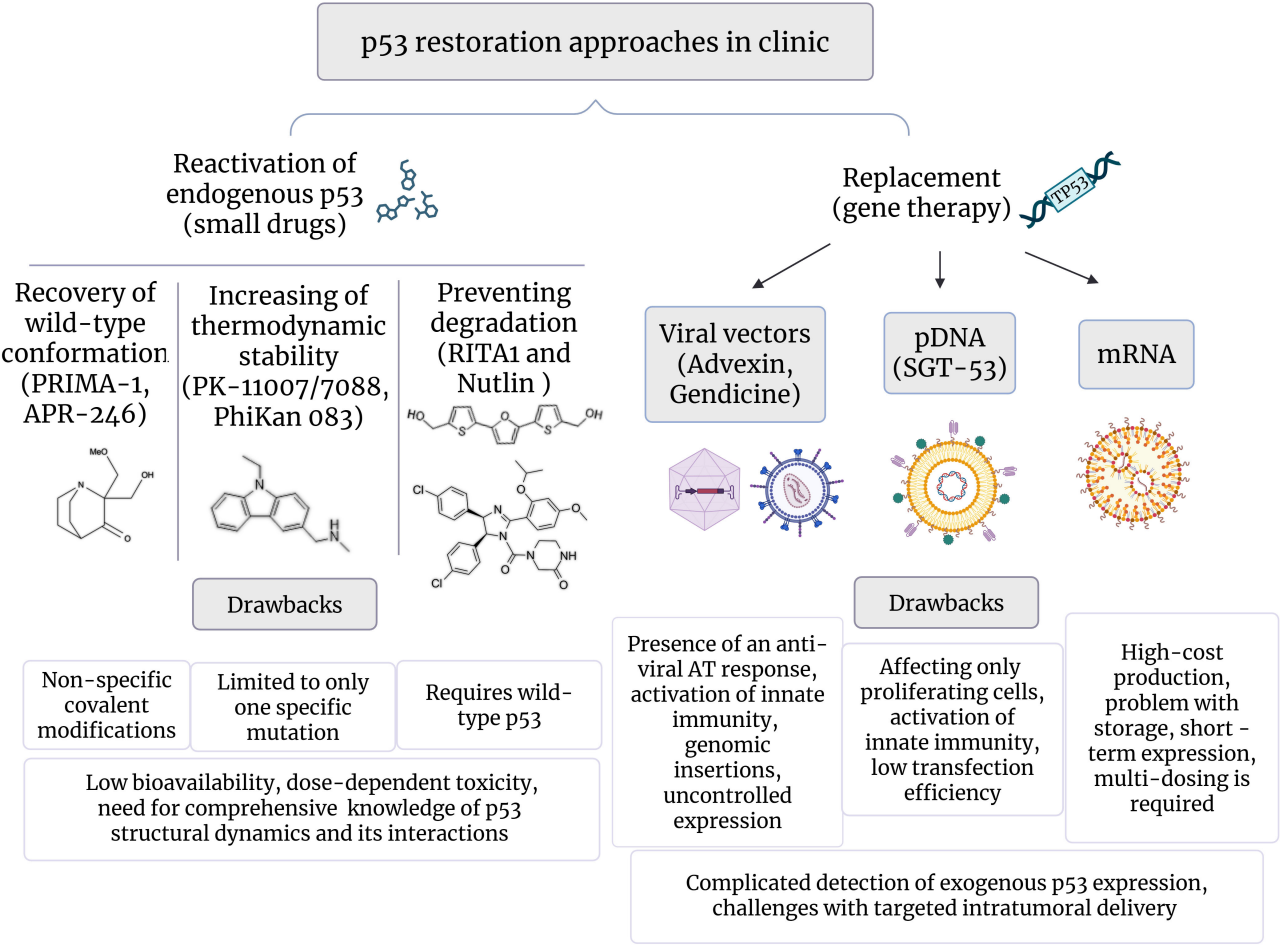
p53 restoration strategies in clinical development. Created in https://BioRender.com.

Small molecule drugs recovering functionality of mutant p53 or modulating activity of wild type protein have emerged as the first prevalent strategy, and a wide range of such therapeutics has been assessed in clinical trials over the past few decades (**Figure 3**); comprehensive analysis is provided in reviews (62–64).

Among them, PRIMA-1 and PRIMA-1Met (APR246, eprenetapopt) have emerged as the first structural reactivators of mutant p53 variants recovering its transcriptional activity by covalent binding cysteines within the core domain (65). In preclinical studies, PRIMA-1 was prospective in rescue of certain p53 mutants, including R175H, R248W, and R273H, however, no clinical benefit was observed (66, 67). APR-246 has been shown to enhance effects of chemotherapy, and it has recently been evaluated in phase 2 and 3 studies in combination treatment regimens [NCT03268382, NCT02999893, NCT03931291, NCT03745716]. Notably, numerous mechanistic studies have revealed p53 independent сytotoxic effects of PRIMA-1 and APR-246, mediated by adduct formation with intracellular glutathione, thus leading to increased reactive oxygen species (ROS) production and triggering oxidative stress (67).

Compounds based on a carbazole scaffold (PK-11007, PK-7088, and PhiKan083) have become an advancement in precision oncology. They rescue p53-Y220C by binding in a specific manner to mutation-induced surface cleft thus preventing opening the cavity and stabilizing p53 native conformation (45, 68). Interestingly, mutant Y220C also demonstrates completely native p53 structure at subphysiological temperature (45). In clinical trials, such drugs provide objective responses and increase median progression-free survival in patients with advanced solid tumors (69). ZMC1 (NSC319726) specifically restores mutant R175H with impaired zinc binding by providing an optimal zinc concentration due to zinc ion-chelating properties (48).

RITA1 and Nutlin exemplify a class of agents designed to reinforce p53 activity by preventing MDM2-mediated degradation (70, 71). RITA1 binds to the N-terminal region of p53, allosterically inhibiting the p53-MDM2 interaction, whereas Nutlin acts as MDM2 antagonist, competing for MDM2’s p53-binding pocket. While both agents have been largely tested across cancers with different p53 status, applicability was mainly confined to tumors bearing wild-type p53 and MDM2 amplification.

Although preclinical and early clinical outcomes were promising, subsequent trial results of small molecule p53 reactivators have been disappointingly modest. This underscores the complexity of p53 regulatory networks in tumor cells, where it remains elusive which specific p53 pathway is essential for tumor suppression. Obviously, to achieve the progress in pharmacological recovering of p53 activity, rational design is essential, which will require comprehensive analysis of both p53 structure dynamics and its interactions with protein partners, as well as realizing how these cooperations are affected by mutation heterogeneity.

### 4.2 Gene replacement therapy

Dysfunctional p53 signaling fails to arrest cell cycle and induce senescence or apoptosis, underlying resistance of p53-deficient tumors to standard chemo- and radiotherapy (72). Interestingly, in an orthotopic mouse model of liver carcinoma with conditionally regulated expression of endogenous p53, restoring p53 activity resulted in complete tumor rejection mediated primarily through induction of cellular senescence and subsequent clearance by the innate immunity (73). Additionally, p53 promotes tumor cell recognition by both innate and adaptive immune systems via up-regulation of miR-34 and miR-200 family, which directly interact with 3’ UTR of PD-L1 mRNA, abrogating immune checkpoint-mediated immunosuppression (74, 75).

Restoration of p53 activity through exogenous TP53 gene expression has become both a treatment concept for p53-deficient cancers and an important milestone in gene replacement therapy (**Figure 3**). In the pioneering clinical trial, conducted in 1996, local delivery of TP53 gene using retroviral vector resulted in partial regression in three of nine NSCLC patients and stable disease in three others (76).

INGN-201 (Advexin) represents a replication-defective adenovirus type 5 vector carrying TP53 under CMV promoter (Ad.5CMV-p53) produced by Introgen Therapeutics Inc (Houston, TX). In a phase I, bronchioloalveolar delivery of INGN-201 lead to stable disease in 16 of 21 patients with BAC, albeit p53 status was not an entry criterion (77). In I/II phase study of intratumoral administration of INGN-201, local antitumor effects were observed in nine of ten patients with chemoresistant esophageal carcinoma, with one had stable disease for 24 months as the best result (78). Perioperative local INGN-201 therapy was tested in a phase II trial of 13 SCCHN patients as post-resection adjuvant treatment of microscopic residual disease (79). Combination of intratumoral INGN-201 and radiation therapy in 19 NSCLC patients yielded complete response in 1 (5%), partial response in 11 (58%), and stable disease in 3 patients (16%) (80). Safety and efficacy of Ad.5CMV-p53 in combination with immune checkpoint inhibitors was evaluated in phase II study conducted by MultiVir, Inc [NCT03544723]. Collectively, Advexin has been tested in multiple trials up to phase III, with relatively safe and positive results, but without FDA approval (81). The persistence of elevated anti-adenovirus IgG titers, complexity of detecting exogenous p53 expression in tumor specimens, and identification of wild-type p53 tumor status as a predictor of clinical response create ambiguity about whether observed antitumor effects are the result of exogenous TP53 expression or immune response to the adenoviral vector (81, 82).

Gendicine, a similar recombinant adenovirus expressing TP53 (rAd5-p53), developed by SiBiono GeneTech Co. (China), was approved by the Chinese Food and Drug Administration for the treatment of SCCHN and brought as the first gene therapy drug authorized for clinical use in China, but not beyond. A detailed review of Gendicine clinical efficacy is provided by Zhang et al. (83).

Nanocomplex-based gene delivery systems have become widely used as a rational alternative to viral vectors, offering an appropriate safety profile, scalability, and versatility for delivering diverse nucleic acid therapeutics. Among them, liposomal, silica-based, and polypeptide nanocarriers have been designed to deliver TP53 selectively to tumor cells (84–86).

SGT-53, developed by SynerGene Therapeutics, represents TP53-encoding plasmid DNA within cationic liposome decorated with scFv of transferrin receptor (TfR) antibody to ensure selectivity for tumor cells based on their overexpression of TfR. SGT-53 potentiated glioblastoma cell line sensitivity to temozolomide (TMZ) *in vitro* and in combination with TMZ drastically inhibited tumor growth in highly TMZ-resistant intracranial GB models (87). An initial phase 1 trial has demonstrated safety of SGT-53 systemic delivery and confirmed the presence of exogenous TP53 DNA in metastatic lesion biopsies (88). In a follow-up phase 1b study, combination of SGT-53 and docetaxel lead to partial responses and stable disease in 3/14 and 2/14 patients, respectively (89). Recent mechanistic studies have shown anti-PD-1 treatment when combined with SGT-53 to provide robust growth control in syngeneic tumor models. Notably, this effect was also reproduced for liposomes loaded with non-coding DNA, while being abrogated in mice lacking CD8+ T cells or STING expression (90). These findings elucidate the activation of STING, a cytoplasmic DNA sensor triggering IFNβ response, as a critical mechanism underlined anti-tumor SGT-53 activity, questioning to what extent the expression of TP53 pDNA contributes to therapeutic effect (90, 91).

The feasibility of recovery p53 activity employing innovative platform based on synthetic mRNA was showed by Kong, designed redox-responsive polymer-lipid nanoparticles (NPs) for efficient tumor-targeted delivery of p53-encoding mRNA. This approach led to restoring p53 expression, sensitizing tumors to sirolimus, and strong antitumor effect in various preclinical HCC and NSCLC models (92). Administration of p53 mRNA within polymer-lipid NPs functionalized with a CXCR4-specific peptide to target murine HCC models reversed immunosuppression in tumor microenvironment (TME) and provided significant tumor growth control in combination with anti-PD-1 therapy (86). Prospectively, innovative mRNA-based platforms open new opportunities for p53-targeted gene therapy, and being integrated with conventional therapeutic regimens, could emerge as a highly effective strategy to treat a wide range of malignancies.

## 5 p53 in the era of cancer immunotherapy

The immunogenicity of p53 epitopes was initially elucidated in 1993 by Melief and al., who confirmed HLA-A02:01-binding capacity for 18 wild-type and 9 mutant p53-derived peptides (93). The authors employed an *in vitro* assay using a TAP2-deficient T2 cell line, which exhibits marked increase in the surface density of HLA class I molecules due to stabilization of HLA upon exogenous loading with high-affinity peptides. The same cell line pulsed with p53 wild type 264–272 and mutant R175H 168–176 peptides was used to generate reactive CTLs *in vitro* from peripheral blood mononuclear cells (PBMCs) of a healthy HLA-A02:01-positive donor (93). In a similar study, Röpke and al. isolated a CTL clone targeting the 264–272 peptide in the context of HLA*A02:01, which was shown to kill cell lines derived from two head and neck carcinomas carrying p53 mutations outside the 264–272 epitope (94).

DeLeo and colleagues first provided proof-of-concept for p53 epitope-based immunization, using dendritic cell (DC)-based vaccine pulsed with the mutant M234I 232–240 peptide, which *in vivo* inhibited growth of chemically induced mouse sarcoma Meth A bearing corresponding mutation (95). Furthermore, a similar vaccine targeting the murine wild-type p53 232–240 epitope resulted in delayed growth of mouse sarcoma CMS4 in pre-immunized mice and rejection of established tumors (95). In a subsequent study, adoptive transfer of CTL clones targeting the murine wild-type 158–166 peptide, isolated from p53^−^/^−^ mice immunized with syngeneic 4J tumor cells overexpressing p53, yielded complete eradication of 4J tumors in nude mice and prevented growth of 5D tumors in immunocompetent mice, without causing immunopathology in normal tissues (96).

The translation potential was underscored by the ability to efficiently generate CTL recognizing wild-type p53 derived 264–272, 149–157 and 65–73 peptides *in vitro* through stimulation T-lymphocytes with peptide pulsed autologous DCs isolated from SCCHN patients and healthy donors (97, 98). Moreover, it was shown that variants of 264–272 peptide with substituting amino acids LLGRNTFEV and LLGRNSWEV can abrogated nonresponsiveness to original 264–272 epitope seen in some donors (99). The expanded CTL clones mediated cytotoxicity against several cancer cell lines, including SCC-9 and MDA-MB-468 (97–99). Similarly, CTL clones targeting HLA-A*0201-restricted mutant epitopes were generated from peripheral blood lymphocytes of SCCHN patients with mutations S149C, Y220C, and Y220H (100).

**Figure 4:**
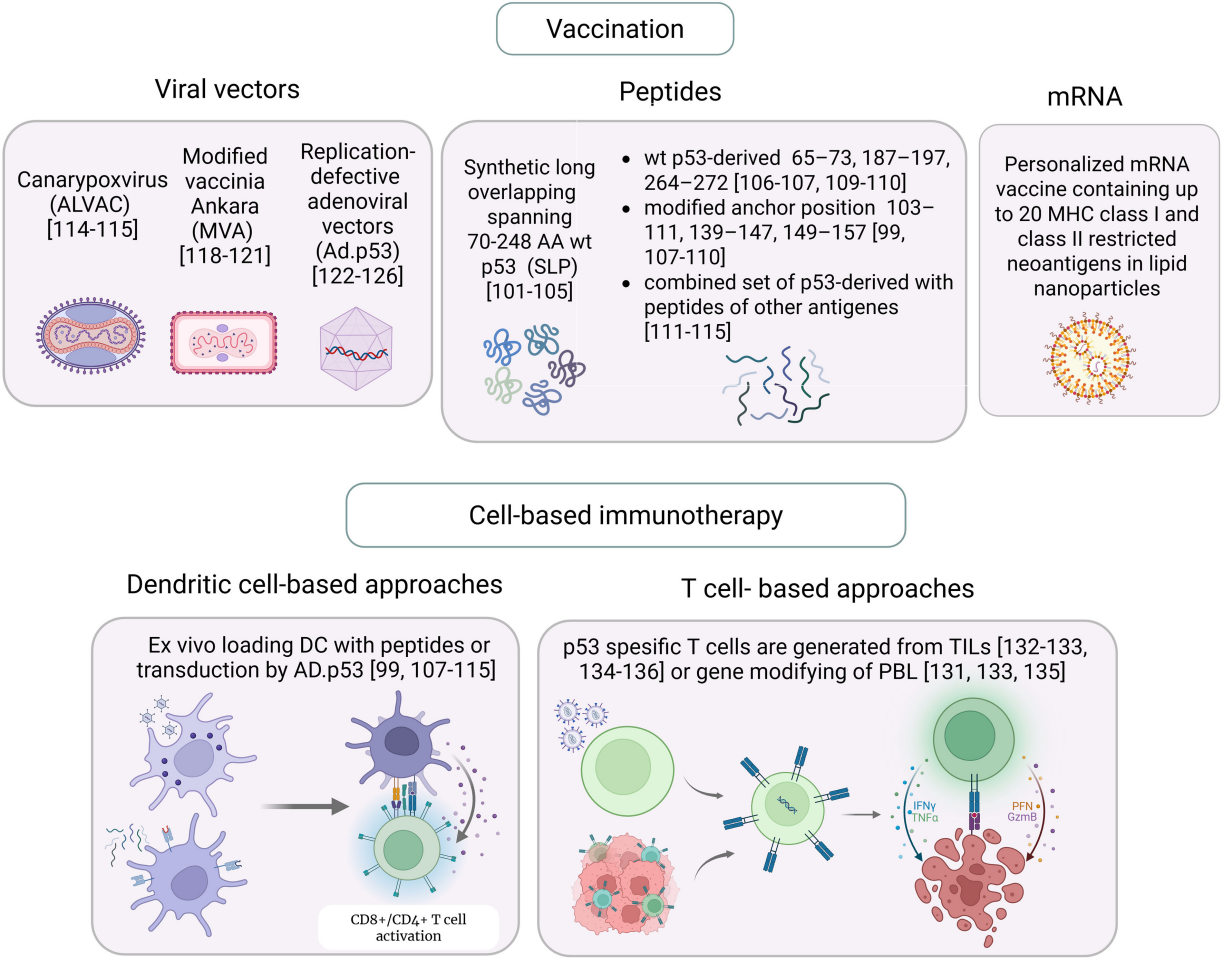
Immunotherapeutic approaches targeting p53 in cancer. Created in https://BioRender.com.

Collectively, these findings indicated the tolerance to self p53-derived peptides *in vivo*, likely resulting from their significantly decreased surface presentation by normal tissues, and established the ability of p53-specific CTLs to selectively eliminate p53-overexpressing tumor cells, highlighting both mutant and wild-type p53 epitopes as attractive tumor-associated antigens for immunotherapeutic strategies (**Figure 4**).

### 5.1 Peptide-based vaccines

Following initial identification of 264–272 peptide as an immunogenic HLA-A2-restricted epitope of wild-type p53, the Melief’s group has systematically explored the complete landscape of p53 antigenic determinants capable to elicit CD8+ T-cell responses. As a result, synthetic long overlapping peptides (SLP) of 20–30 amino acids spanning 70–248 wild-type p53 residues were designed and being evaluated in clinical trials as therapeutic anticancer peptide vaccines, which can be used irrespective of patient HLA alleles, enabling processing into a range of epitopes.

In a phase I/II study, ten patients with metastatic CRC were immunized subcutaneously (SC) with p53-SLP using Montanide ISA-51 as an adjuvant (101). p53-specific T-cell responses were detected for nine of ten patients with persistent T-cell reactivity for at least 6 months, however all responses were restricted to CD4+ T-cell subset. The vaccine was well-tolerated, without toxicity exceeding grade 2. In a follow-up trial involving nine CRC patients, the same SLP vaccine combined with IFN-α induced p53-specific CD4+ T cells with enhanced IFN-γ production across all patients and generated a CD8+ T-cell response in one patient (102).

In a phase II trial, 18 patients with recurrent ovarian cancer (ROC) immunized with p53-SLP developed p53-specific T-cell responses, confined to CD4+ T cells (103). Although two patients achieved stable disease as the best clinical effect, long-term follow-up didn’t reveal increase in response rate to chemotherapy nor statistically significant difference in survival (103). Subsequently, ten patients with ROC were pretreated with low-dose cyclophosphamide to suppress regulatory T cells (Tregs) and enhance the clinical efficacy of p53-SLP vaccine. While the protocol did not decrease CD4+FoxP3+ T-cell levels, it prevented rapid post-immunization decline of circulating vaccine-induced p53-specific T cells and resulted in stable disease in 20% of patients (104). The regimen combining gemcitabine, IFN-α and p53-SLP vaccine therapy induced p53-specific CD4+ Th1/Th2 T cells with enhanced reactivity *in vitro*, achieving two partial responses (PR) and four stable disease (SD) outcomes in 16 patients (105).

Herrin et al. evaluated the optimal way for vaccine delivery of the 264–272 peptide in patients with recurrent ovarian cancer. In arm A, 9 of 13 (69%) patients treated SC with the peptide mixed with Montanide and GM-CSF, and in arm B, 5 of 6 patients (83%) receiving intravenous infusion of autologous peptide-loaded DCs, developed p53-specific CD8+ T cell responses. The median overall survival was 40.8 months (arm A) and 29.6 months (arm B), with median progression-free survival of 4.2 and 8.7 months, respectively (106).

Schuler et al. assessed the clinical efficacy of autologous DCs loaded with 264–272 (F270W) (99) and 149–157 (T150L) (107) modified peptides as adjuvant therapy in 16 patients with HNSCC (108). Post-vaccination, p53-specific CTLs were detected in 11 patients (69%) with no grade II–IV adverse events, achieving a two-year disease-free survival of 88% versus 50–70% in historical controls. Despite the vaccine’s benefits, the authors noted that the employed dendritic cell generation protocol yielded DCs with compromised phenotype common in HNSCC patients, proposing an optimized protocol to enhance DC maturation (108).

Svane et al. conducted a phase I trial of autologous DCs loaded with a set of three wild-type (65–73, 187–197, 264–272) and three modified (103–111, 139–147, 149–157) peptides, in which the anchor P2 position was replaced with leucine to enhance HLA-A2-binding affinity. Of six patients with metastatic breast cancer who received at least eight immunizations, four showed increased p53-specific T-cell responses, with stable disease achieved in two patients (109). In a phase II extension study of 19 patients with advanced breast cancer, eight showed progression-free survival or partial regression, with the median survival 13.8 months in responders versus 4 months in non-responders (110).

In subsequent trials, the vaccine used six tested p53-derived peptides was extended to include survivin- and telomerase-derived peptides (111–113). In a phase I/II study, 36 patients with advanced melanoma were treated with multi-antigen peptide-loaded DCs, IL-2, and IFN-α, achieving stable disease in 6 (17%). A marked increase in CD4+CD25highFoxp3+ Tregs were noted in progressive patients (114). In a phase II study, 28 patients with metastatic melanoma received peptide-loaded DCs combined with cyclophosphamide and celecoxib to achieve Treg reduction. Stable disease was observed in 16 (57%), exceeding 7 months in 8 (22%) patients (115). The authors affirmed the therapy to yield favorable clinical outcomes, with potential for greater efficacy in patients with early-stage disease (110, 115).

Despite dendritic cells have consistently been demonstrated to induce antigen-specific T-cell responses *in vivo*, the key challenge in clinical settings remains a generation DCs with a fully immunocompetent phenotype. Suboptimal DCs functionality stems from both compromised immunity of patients with advanced cancer and imperfect protocols for DC maturation and peptide loading *in vitro*. To address these limitations, rational phenotypic markers of vaccine-grade DCs must be established.

### 5.2 Viral vector-based vaccines

Several recombinant viral vector platforms were explored as anti-cancer vaccines targeting tumors with aberrant p53 signaling, including those harboring mutant TP53 alleles. These platforms encoding full-length p53 aim to elicit antigen-specific T-cell responses and provide a range of p53 epitopes for both CD4+ and CD8+ T-lymphocytes thus bypassing patient-specific MHC restrictions. Moreover, a supportive inflammatory context, triggered by viral components, potentially engage the synergy of innate and adaptive immunity against p53-driven malignancies (116).

A recombinant canarypoxvirus (ALVAC) vaccine encoding wild-type p53 was assessed in a phase I/II study in 15 patients with p53-overexpressing metastatic colorectal cancer (117). No unwanted auto-immune reactions were observed. IgG and T cell responses against ALVAC were detected in all patients except one. p53-specific T cell responses were induced in two patients in the full-dose group, with one achieving stable disease after repeated immunization (117).

Modified vaccinia virus Ankara (MVA), approved by FDA as a smallpox vaccine vector, was adapted to deliver TP53 gene and assessed in a pilot study, involving eight patients with refractory gastrointestinal cancers (118). All high-dose patients developed pronounced p53-specific CD8+ T cell responses, which diminished after repeated doses, but could be restored by PD-1 antibody blockade *in vitro*. In contrast, increasing p53 reactivity in the CD4+ subset did not reach statistical significance. Despite robust immunological responses, no clinical responses were observed per RECIST criteria (118). In a phase I trial of p53MVA vaccine combined with gemcitabine in patients with platinum-resistant ovarian cancer, post-therapy expansion of p53-specific CD4+ and CD8+ T cells was observed in 5 of 11 and 6 of 11 patients, respectively, which achieved a statistically significant extension of progression-free survival (119). A combination of pembrolizumab and p53MVA vaccine used in patient with triple-negative breast cancer led to complete clinical response, marked by cutaneous metastasis regression, which maintained for six months and correlated with persistent p53-specific CD8+ T-cells in peripheral blood (120). In a follow-up study involving 11 patients with advanced solid tumors, three achieved stable disease lasting 30, 32, and 49 weeks, respectively (121).

A recombinant replication-defective adenoviral vector (AV) encoding full-length TP53 (rAd/hup53) was evaluated in six advanced-stage cancer patients. While all participants developed anti-adenoviral humoral and cellular immune responses, vaccination failed to elicit p53-specific T-cell reactivity (122).

In a pilot study of DCs transduced with similar adenovirus (Ad.p53-DC) for therapy of advanced small cell lung cancer (SCLC), p53-specific T-cell responses were developed in 16 of 28 patients (123). One patient achieved a partial response, and seven maintained SD. Subsequently, objective clinical responses were observed in 61.9% of the 21 patients treated with second-line chemotherapy immediately after vaccination, remarkably exceeding historical controls (<16%) (123). In a phase II trial, Ad.p53-DC was used in combination with all-trans-retinoic acid (ATRA) to inhibit myeloid-derived suppressor cells. p53-specific responses were observed in 0%, 20%, and 41.7% of SCLC patients in arm A (observation), B (Ad.p53-DC), and C (Ad.p53-DC plus ATRA), respectively (124). In an extended trial, overall response rates (ORRs) to the second-line chemotherapy were 15.4%, 16.7%, and 23.8% for arms A, B, and C, with no survival differences between arms (125). Ad.p53-DC vaccine combined with indoximod assessed in 39 patients with metastatic breast cancer, elicited p53-specific T-cell expansion in 30%, with SD as the best clinical outcome in four patients (126).

While viral vectors mediate effective transgene delivery into antigen-presenting cells (APCs) with robust activation of innate immune pathways (116, 127), the clinical benefits of these vaccines remain insufficient. One reason is that immunodominant epitopes of viral carriers may be prioritized during antigen presentation, overwhelming the priming of p53 specific T-cells (116, 118). Along with it, p53-specific T-cell responses generated after the first vaccination fail to expand following repeated immunizations, likely due to increased levels of antiviral antibodies, which markedly reduce the efficacy of viral vector-based vaccines (116, 128, 129).

### 5.3 Adoptive TCR-T therapy

*In vitro* expansion of CTL clones, which target tumor-specific or tumor-associated antigens, derived from autologous T lymphocytes, followed by patient reinfusion, could address limitations of suboptimal T-cell responses elicited by vaccination, as well as anergy and exhaustion of endogenous antigen-specific CTLs caused by immunosuppressive TME and chronic antigenic stimulation.

The therapeutic potential of targeting cells with aberrant p53 signaling through adoptive cell transfer (ACT) of T lymphocytes recognizing p53-derived epitopes was initially demonstrated *in vivo* by McCarty et al. (130). High-affinity CTLs specific for human p53 149–157 peptide were generated in transgenic mice expressing HLA-A*02:01 allele and shown to selectively lyse p53-overexpressing HLA-A*02:01+ pancreatic carcinoma cell line Panc-1, but not normal fibroblasts or HLA-A*02:01- tumor cells. Adoptive transfer of isolated CTLs significantly reduced the growth of Panc-1 xenograft in SCID mice, with infiltration observed in regressing tumors (130).

In a study pioneered by Rosenberg, human peripheral blood lymphocytes (PBLs) were engineered via retroviral gene transfer to express a murine TCR specific to HLA-A*02:01-restricted p53 264–272 epitope (131). These modified T cells selectively recognized and killed various HLA-A*02:01+ p53-overexpressing tumor cell lines, as well as fresh tumor cells, underscoring that T lymphocytes with genetically engineered p53-specific TCR hold promise for adoptive targeting a broad range of malignancies.

Subsequently, PBLs were genetically modified with a retroviral vector encoding a humanized TCR targeting p53 264–272 peptide for use in a phase II trial involving patients with p53-overexpressing metastatic cancer [NCT00393029]. Similarly, a treatment regimen combining anti-p53 TCR-modified PBLs, aldesleukin and Ad.p53-DC vaccine was evaluated for its ability to achieve tumor regression [NCT00704938].

Preclinical studies over the past few years in the Rosenberg laboratory have focused on targeting mutant p53 neoantigens for ACT cancer immunotherapy. Specifically, TCR profiling of tumor infiltrated lymphocytes (TILs), derived from metastases of two ovarian cancer patients with p53 hotspots G245S and Y220C, identified TCRs recognizing respective neoantigens (132). These TCRs mediated reactivity in the DRB3*02:02 context and selectively recognized mutated peptides, as evidenced by 4-1BB expression in co-cultures with autologous DCs pulsed with mutated but not wild type peptide. Three TCRs specific for p53 neoepitope HMTEVVRHC were isolated from TILs of patient with CRC harboring p53 R175H (133). Retrovirally transduced into allogeneic PBLs, these TCRs mediated A*02:01-restricted recognition of ovarian cancer, uterine carcinoma, myeloma, and esophageal adenocarcinoma cells with endogenous p53 R175H expression. Given the frequency of R175H, Y220C, and G245S p53 found in human cancer (5.5%, 2.8%, and 1.6%, https://cancer.sanger.ac.uk/cosmic) and prevalence A*02:01 and DRB3*02:02 alleles (30-40%, http://www.allelefrequencies.net), it is estimated that nearly 4% of cancers patients all over the world could benefit from ACT using isolated TCRs (43, 132, 133).

A TP53-specific assay based on tandem minigenes and pulsed peptides was developed to extend the immunogenicity screening to 12 TP53 hotspot mutations (134, 135). Remarkably, TILs from 11 of 28 (39%) analyzed epithelial tumors with the 8 most frequent p53 mutants responded when co-cultured with DC pulsed with autologous mutated p53 epitopes. As a result, nine TCRs specific for seven unique p53 neoepitopes, restricted by a range of HLA alleles, were characterized. Both isolated TILs and TCR-engineered allogeneic T cells showed reactivity against tumor cell lines bearing corresponding HLA and TP53 allele (134). Subsequently, the library of p53 neoantigen-specific TCRs was extended to 39 TCRs with 21 unique reactivities encompassing TP53 mutations accounting for 7.3% of patients with solid tumors (135).

The same screen approach was employed to show that p53 neoantigen-specific T cells are present in antigen experienced T lymphocytes from PBLs of all patients who had intratumoral p53 TIL responses and absent in those lacking this TIL reactivity. As a result, p53 neoantigen-specific TCRs isolated from TILs and PBLs were identical in all but one donor, indicating circulating peripheral blood T cells as a promising source of p53 neoepitopes-reactive T cells and TCRs for ACT - a finding that is especially important for patients ineligible for surgery (136).

Clinical application of adoptive cell transfer using autologous T cells derived from expanded TILs or TCR-engineered PBLs, which recognize unique or public tumor neoantigens, has garnered interest in the past 10 years (137). In two clinical trials evaluating expanded TP53 neoantigen-reactive TILs for the treatment of 13 patients with chemorefractory epithelial cancers, only three partial responses were observed, likely due to the exhausted phenotype and low levels of mutant p53 reactivities in infused cell products (133, 135). In contrast, one patient who received PBLs genetically modified by TCR targeting p53 R175H achieved a tumor regression lasting 6 months (135). Noteworthy, protocols for generating neoantigen-specific TCRs are actively being optimized as evidenced by the first complete durable responses, reported in advanced cancer patients (138–141).

## 6 Conclusion

Over four decades since its discovery, profound insights into p53’s role in cellular homeostasis and its regulatory activities have been acquired, inspiring discoveries in multiple aspects of cell biology, including genetic and epigenetic stability, cell death, senescence, various types of stress responses, metabolic regulation, and oncogenesis. Our comprehension of p53 has evolved from its initial identification as an oncogene to re-profiling in the “genome guardian”, and finally to current status of the master regulatory factor in numerous cellular pathways. Notably, there is still no consensus on whether p53 mutants act as active oncogenes and to what extent their aberrant activity is essential for maintaining the malignant cell phenotype in established malignancies. The complexity of p53 regulatory networks in both normal and tumor cells, as well as its high structural plasticity, pose significant challenges for pharmacological targeting.

Concurrently, p53 has proven to be a rational target for cancer immunotherapy, with mechanisms underlying the ability of both mutant and wild-type p53-derived antigens to render robust and selective immune targeting of malignant cells. In the last twenty years, different p53-based vaccination approaches, including viral vectors, dendritic cells, short and long peptides, have been tested for the treatment of p53-overexpressing cancers. Despite established safety and the capacity to elicit p53-specific immune responses, the clinical benefits of p53 vaccines have generally been modest. Adoptive cell transfer with T lymphocytes genetically engineered to express TCRs specific for p53 neoantigens emerge as effective personalized therapy, promising to address the challenges of conventional p53-based vaccinations and achieve sustained tumor regression of p53-driven malignancies.
